# Supplementary material for: Single-cell analysis reveals cellular heterogeneity and limits of marker-based assessment in retinal ganglion cell-enriched organoid cultures
Source: Mol Med. 2026 Apr 30;32:103. doi: 10.1186/s10020-026-01488-3 (PMC13339495; doi:10.1186/s10020-026-01488-3)
Supplement: Supplementary file 1 — Supplementary Material 1. [file 10020_2026_1488_MOESM1_ESM.docx]

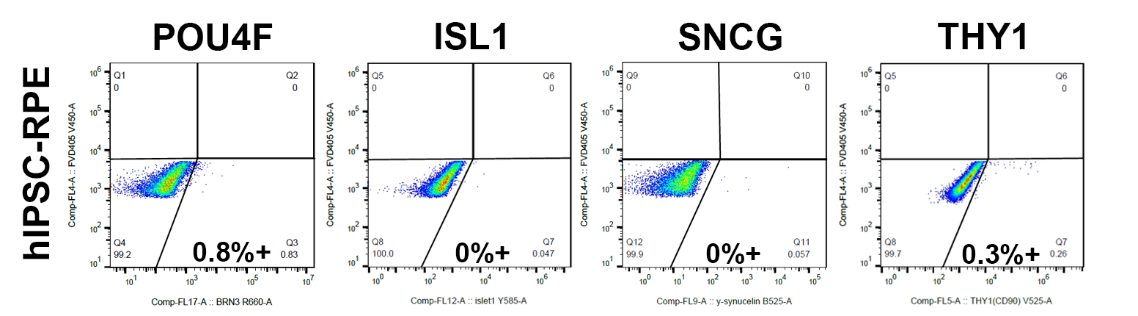


**Figure S1. Negative control for flow cytometry assessment.** hPSC-derived RPE was used as a negative control for the antibodies POU4F, ISL1, SNCG, and THY1.


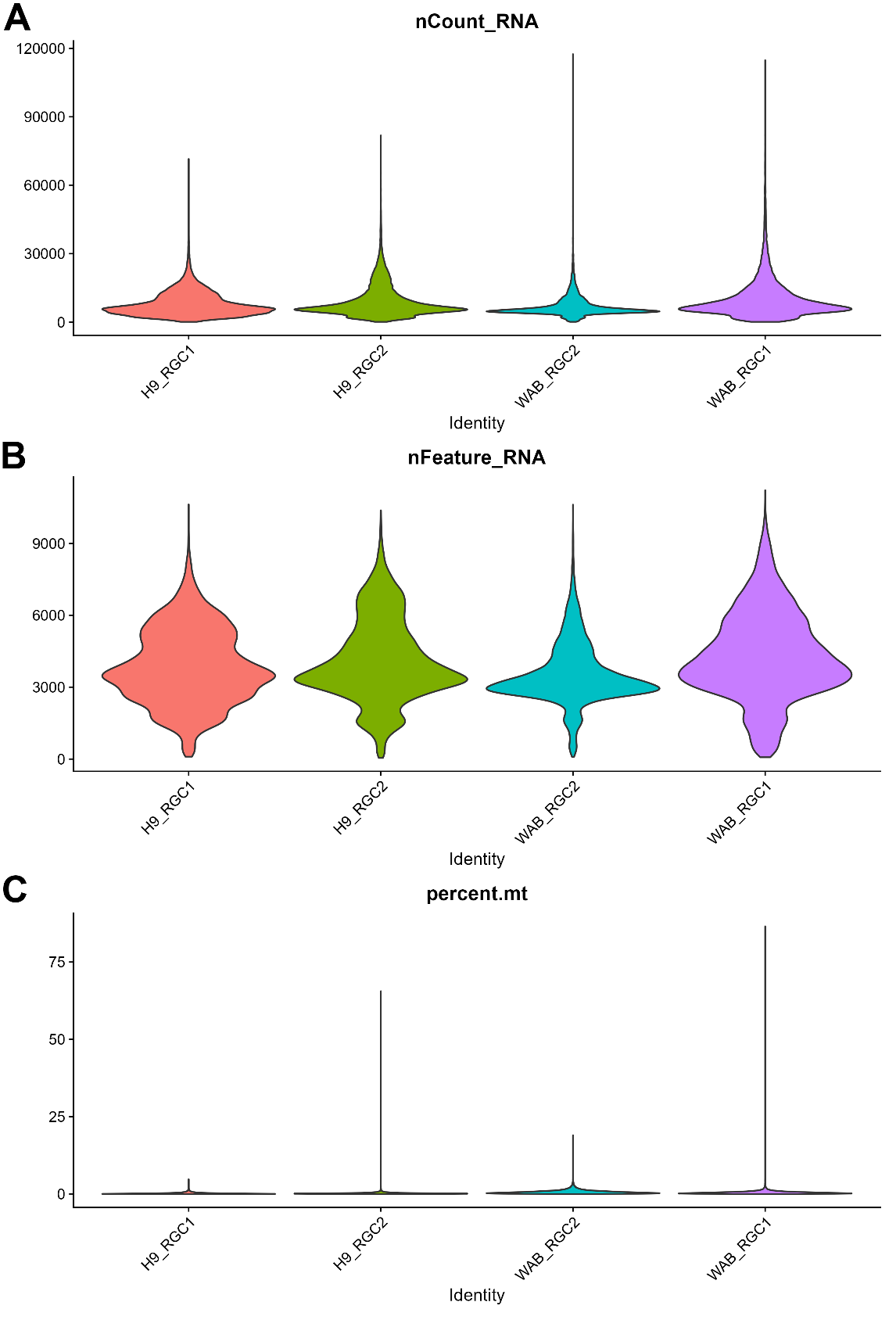


**Figure S2. Pre-filtering quality-control metrics.** Violin plots showing **(A)** total RNA counts per cell (nCount_RNA), showing the distribution of UMI counts across samples. **(B)** Number of detected genes per cell (nFeature_RNA). **(C)** Percentage of mitochondrial transcripts (percent.mt).


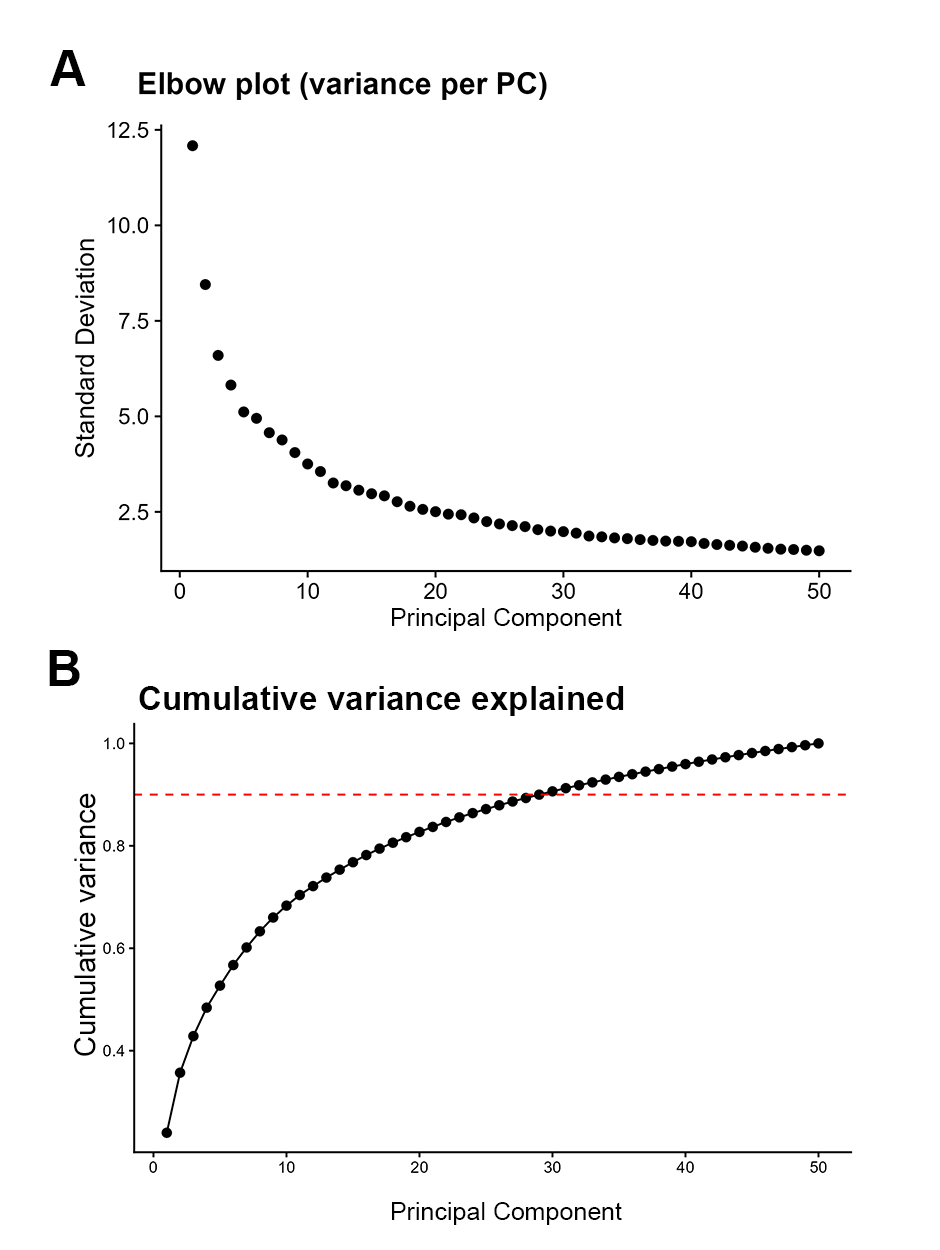


**Figure S3. Principal component selection for downstream single-cell analysis** (**A**) Elbow plot showing the point at which additional principal components (PCs) contribute minimal explanatory power. An inflection is evident at around PCs 20-25, indicating that the dominant biological structure is captured within this range. (**B**) Cumulative variance explained across PCs, with the 90% cumulative variance threshold reached at approximately PC30 (dashed line).


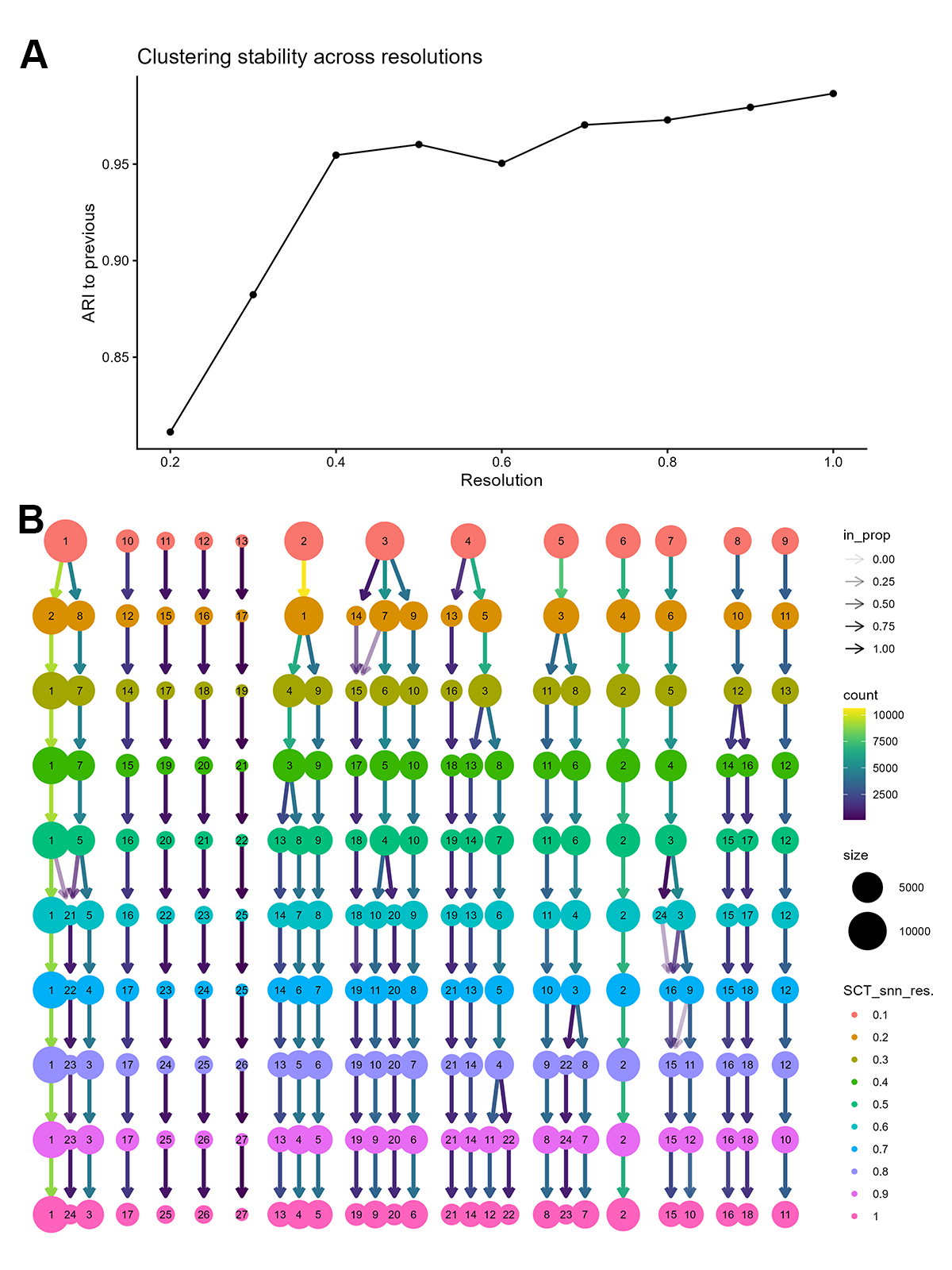


**Figure S4. Determination of clustering resolution based on ARI profiles and clustree analysis.** (**A**) Adjusted Rand Index (ARI) relative to the previous resolution, showing that cluster assignments stabilise at intermediate resolutions, with a clear plateau around resolution 0.5. (**B**) Clustree visualisation illustrating cluster propagation and stability across increasing resolutions across resolutions.


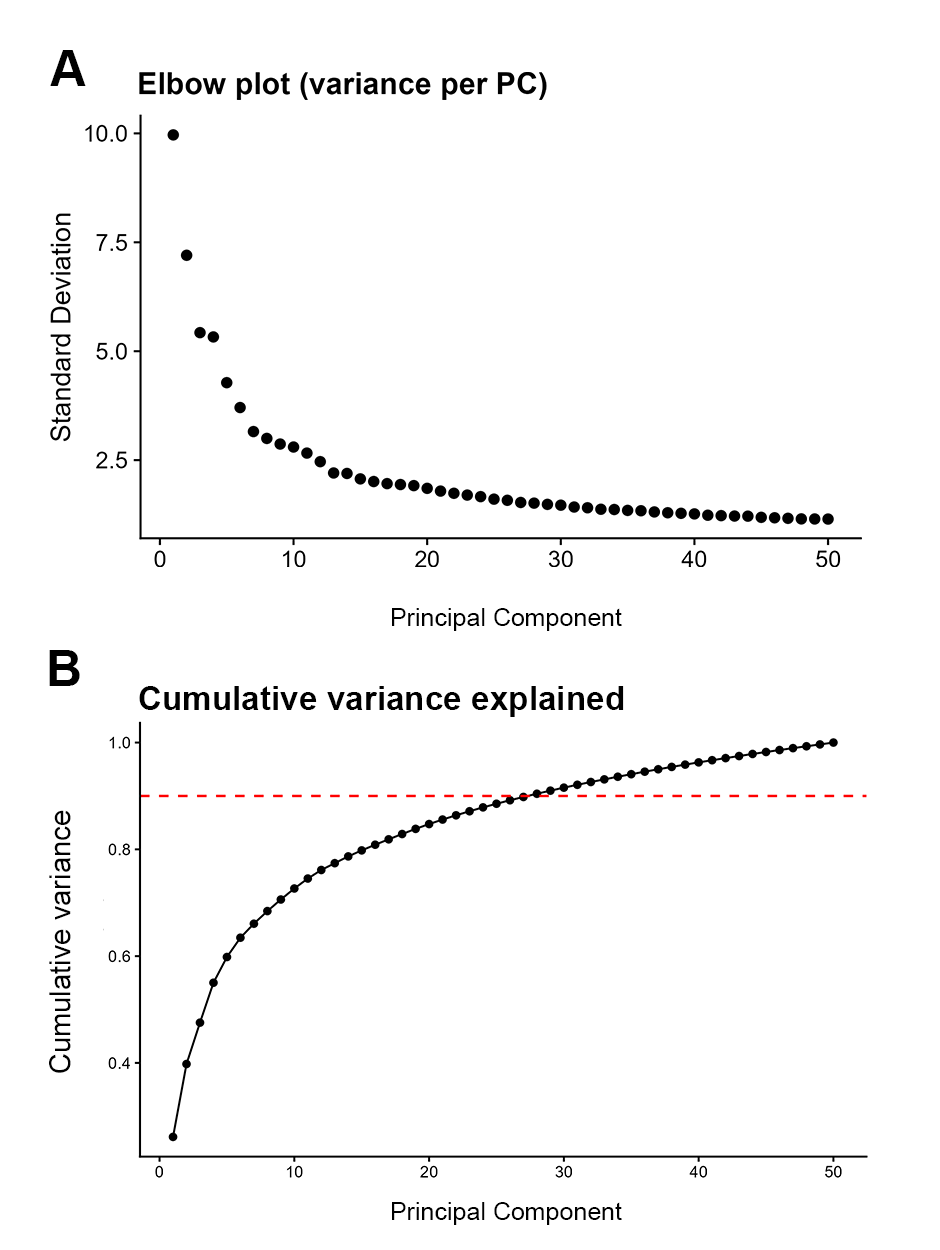


**Figure S5. Principal component selection for subclustering of RGC populations.** (**A**) Elbow plot showing the point at which additional principal components (PCs) contribute diminishing explanatory power, with an inflection observed around PC15. (**B**) Cumulative variance explained across PCs. Dashed horizontal lines indicate the 90% cumulative variance threshold used to guide PC selection.


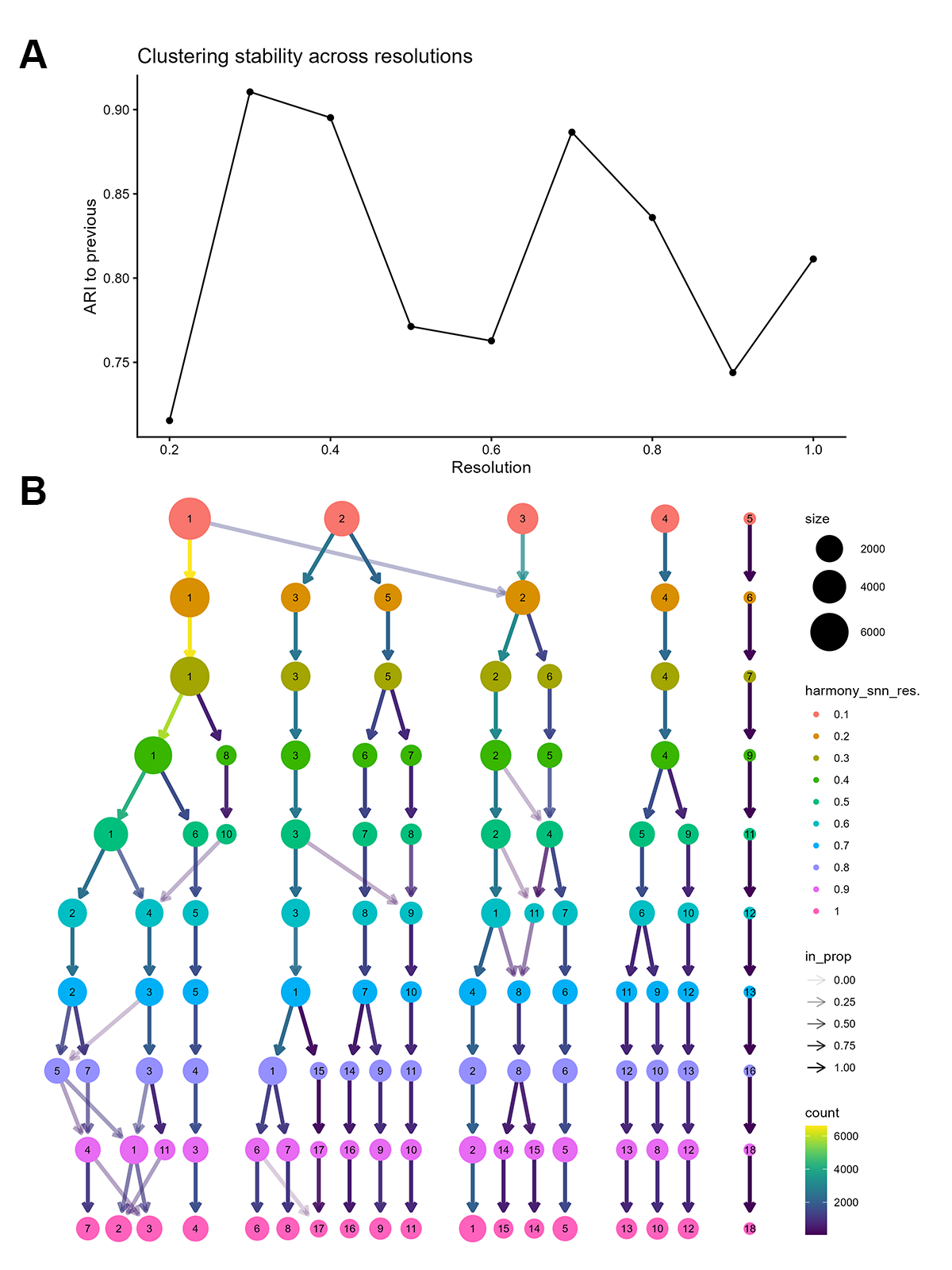


**Figure S6. Determination of clustering resolution based on Adjusted Rand Index (ARI) profiles and clustree analysis for the subclustering of RGC populations. (A)** The ARI-to-previous plot shows that cluster assignments reach their highest stability at around resolution 0.3. (B) Clustree visualisation illustrating cluster propagation across resolutions.

**
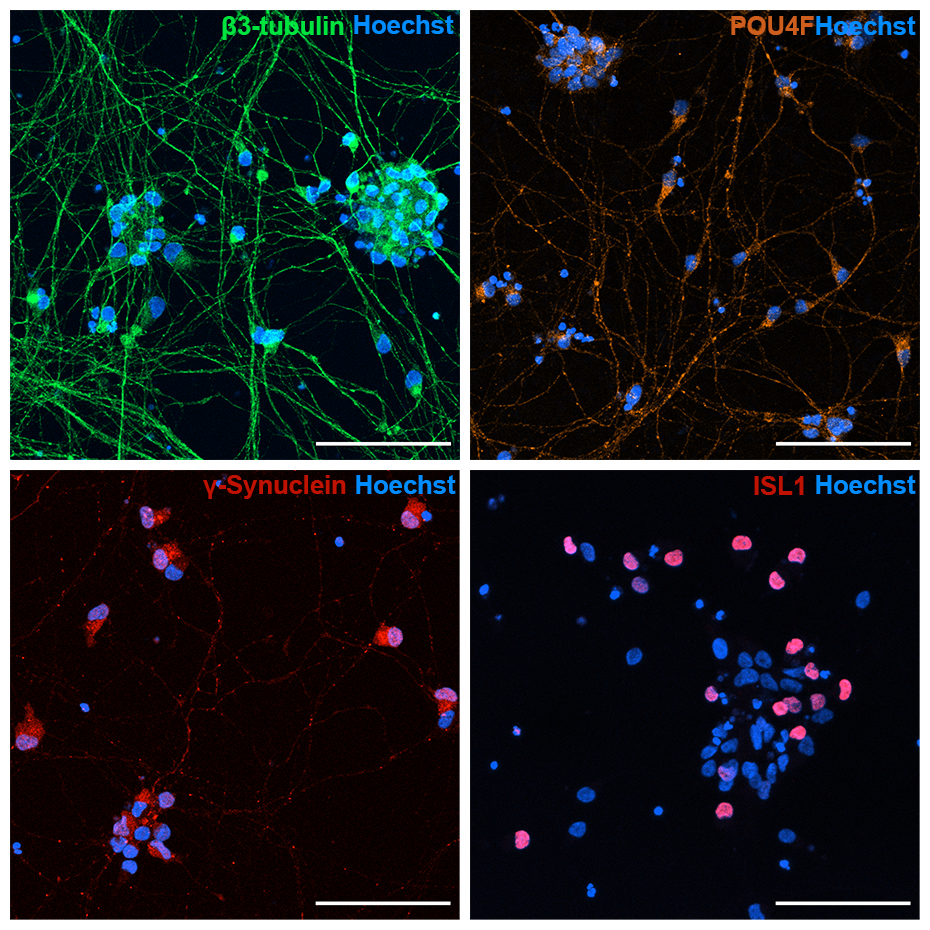
**

**Figure S7. Immunofluorescence characterisation of RGC-enriched cultures derived from H9_RGC1.** Representative immunofluorescence images of cells following 14 days of RGC enrichment after organoid dissociation. Neuronal networks are visualised by β3-tubulin (green), highlighting extensive neurite outgrowth. RGC identity is further supported by expression of canonical RGC markers POU4F (orange), γ-synuclein (red), and ISL1 (red). Nuclei are counterstained with Hoechst (blue). Scale bars: 100 μm.


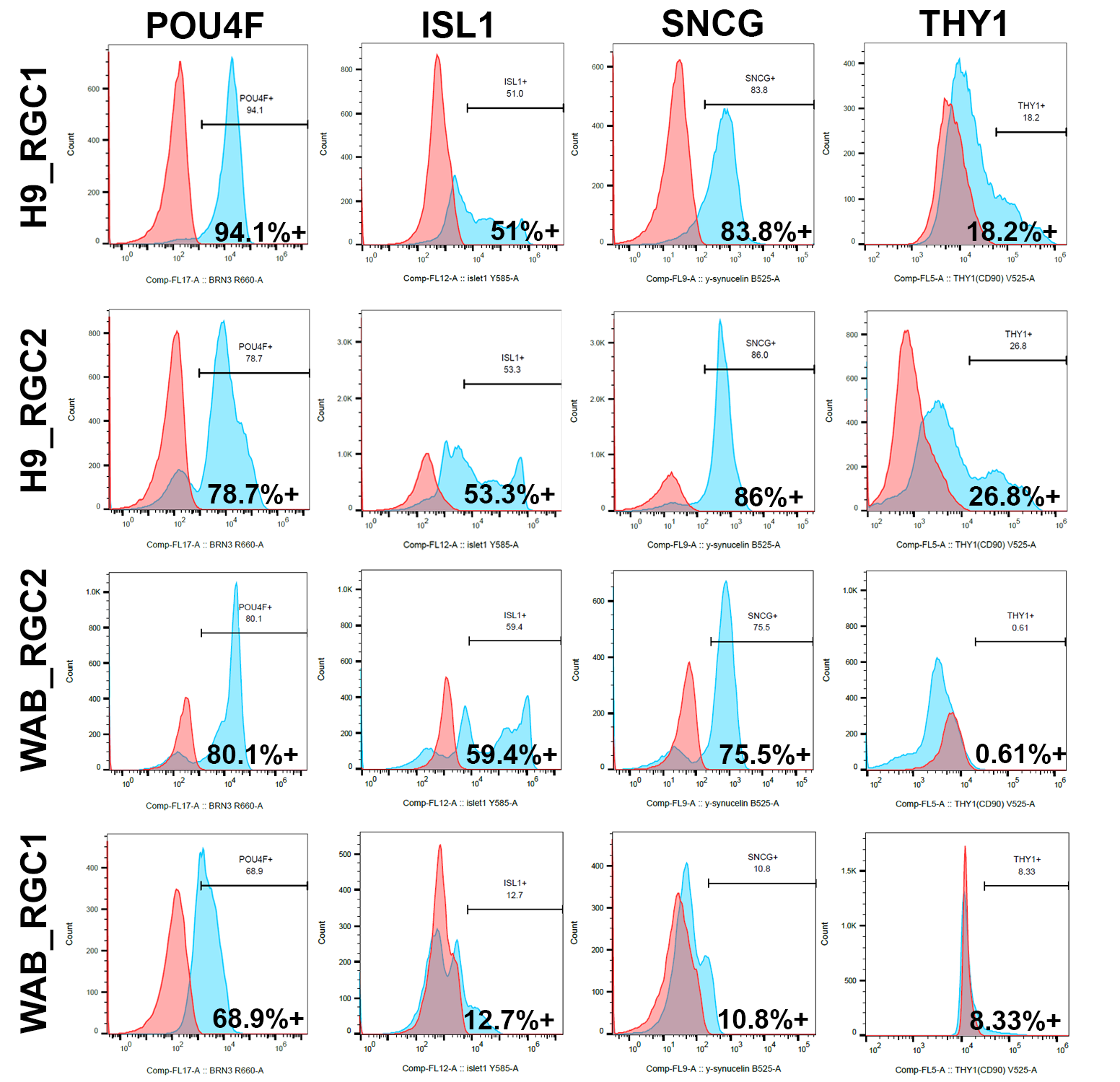


**Figure S8. Histogram representation of RGC marker expression measured by flow cytometry.** Representative fluorescence intensity histograms showing protein-level expression of POU4F, ISL1, SNCG, and THY1 across H9_RGC1, H9_RGC2, WAB_RGC2, and WAB_RGC1. Histograms depict the distribution of signal intensity within the live singlet population, with marker-positive fractions defined relative to unstained controls. Percentages indicate the proportion of marker-positive cells for each sample and marker.


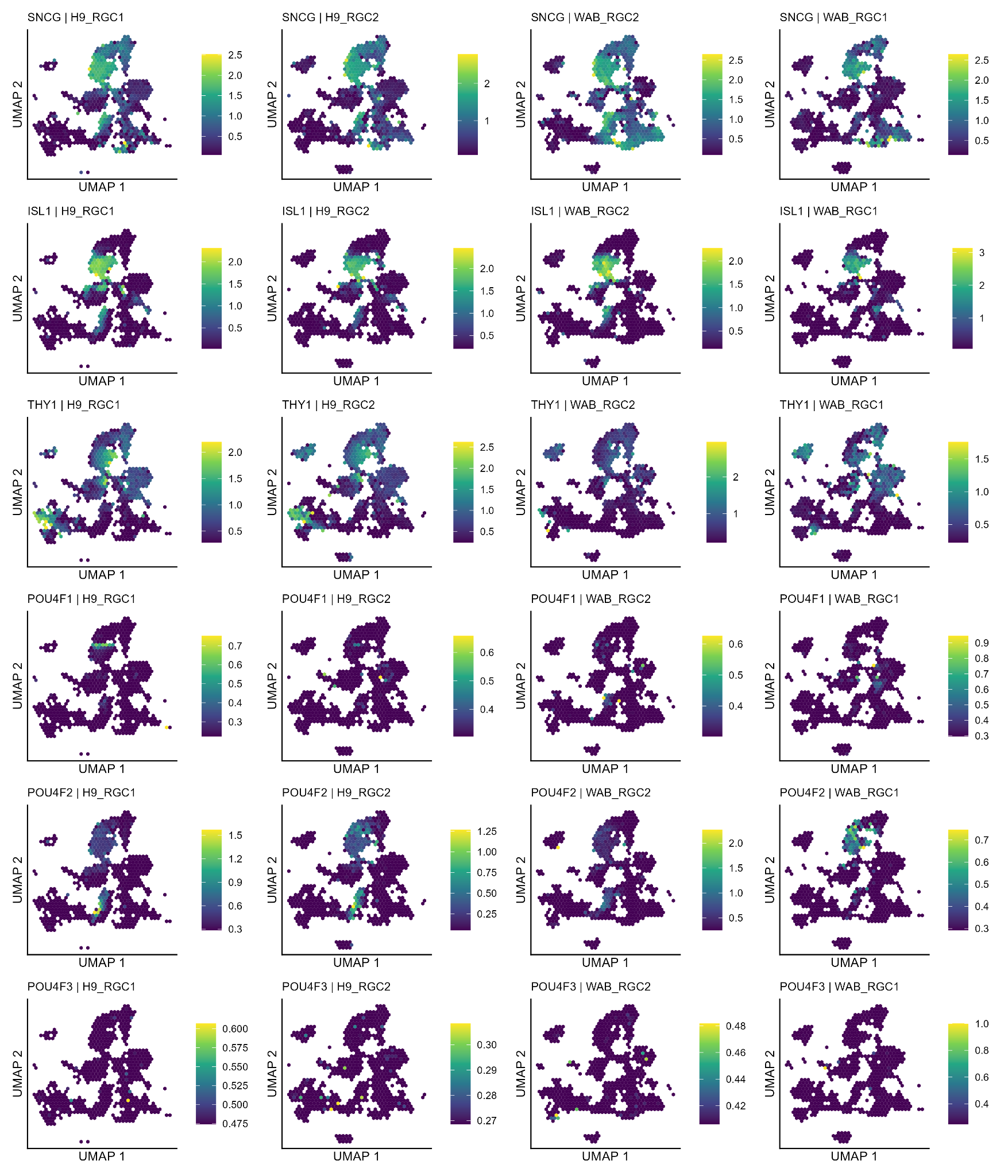


**Figure S9. Spatial distribution of RGC marker expression visualised using hexagon-binned UMAPs (schex).** Hexagon-binned UMAP visualisations showing mean log-normalised RNA expression of canonical RGC markers across the integrated scRNA-seq dataset, displayed separately for each sample (H9_RGC1, H9_RGC2, WAB_RGC2, and WAB_RGC1). Cells were aggregated into hexagonal bins on the UMAP embedding, and colour intensity represents the mean log-normalised expression across cells within each bin.


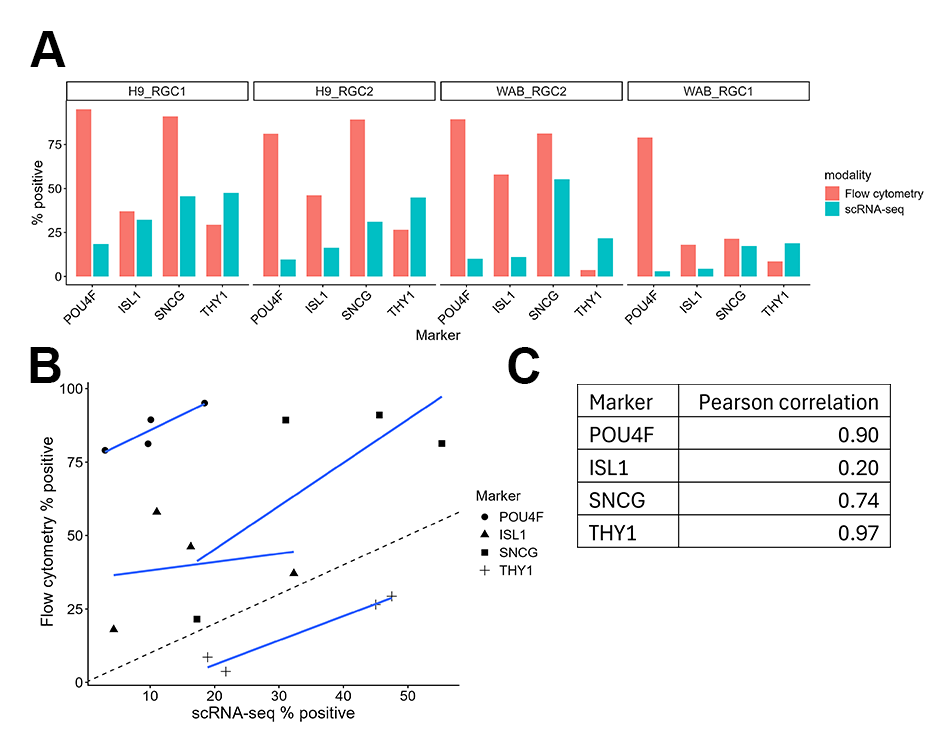


**Figure S10. Quantitative comparison between flow cytometry and scRNA-seq marker detection. (A)** Marker-matched comparison of positive cell proportions measured by flow cytometry and scRNA-seq across H9_RGC1, H9_RGC2, WAB_RGC2 and WAB_RGC1. For scRNA-seq, a cell was considered positive when the SoupX-adjusted RNA count for the corresponding gene was greater than zero. For the pan-POU4F antibody used in flow cytometry, transcript positivity was defined as detection of any POU4F family member (POU4F1, POU4F2 or POU4F3). (**B**) Scatter plot comparing flow cytometry scRNA-seq positivity across all marker-sample combinations. Each point represents one marker in one sample. The dashed line indicates the identity line (y = x), and blue lines represent linear regression fits. Most points fall above the identity line, indicating higher positivity detected by flow cytometry relative to scRNA-seq, except for THY1. (**C**) Summary statistics for marker-matched comparisons between flow cytometry and scRNA-seq detection. Pearson correlation coefficients quantify the relationship between modalities across samples, with higher values indicating stronger concordance.


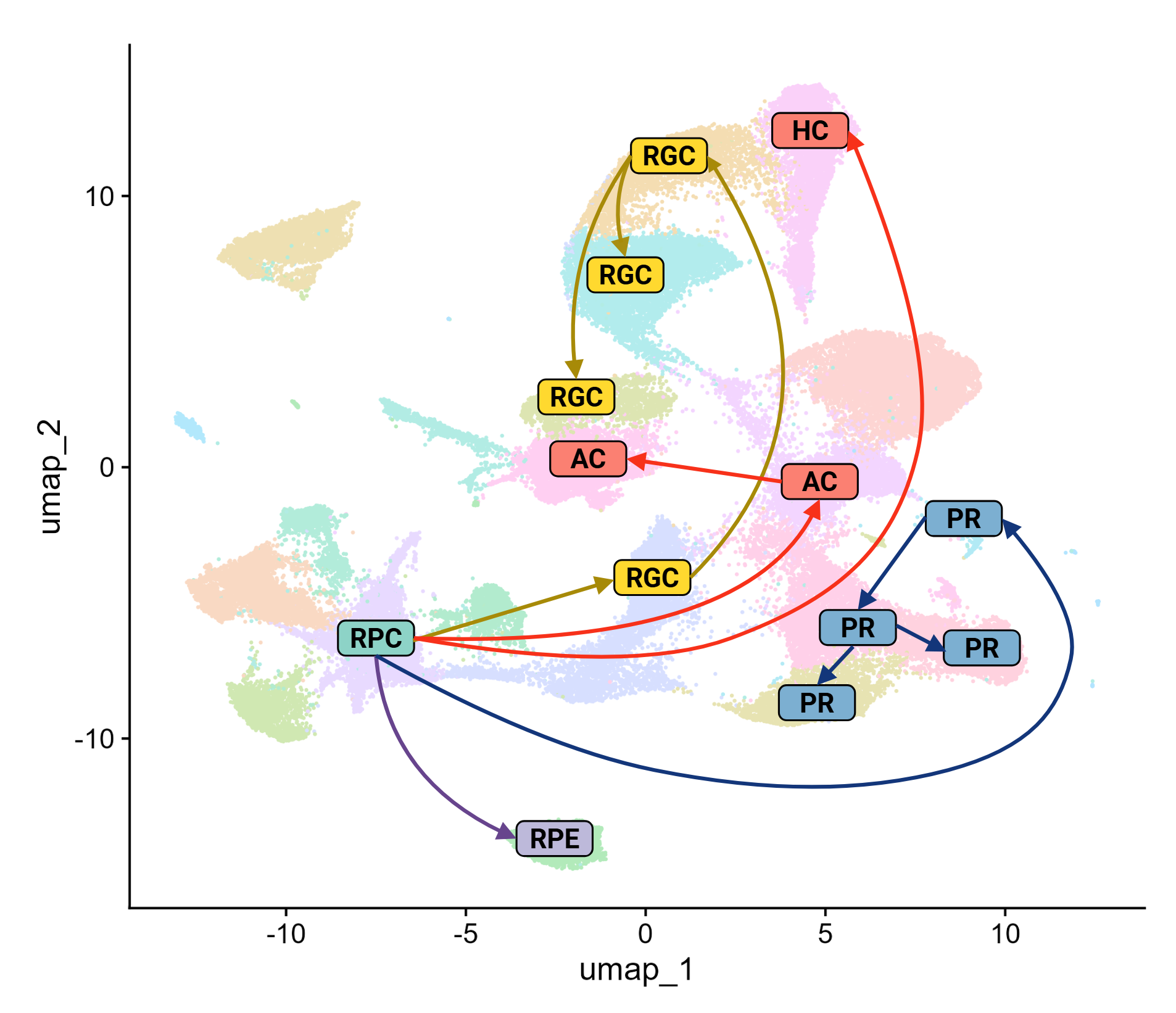


**Figure S11. Pseudotime-inferred progression of retinal lineages visualised on UMAP.** UMAP embedding of the integrated single-cell dataset with arrows indicating the inferred developmental progression of major retinal lineages based on pseudotime analysis. Arrows illustrate transitions from retinal progenitor cells toward differentiated retinal cell types.


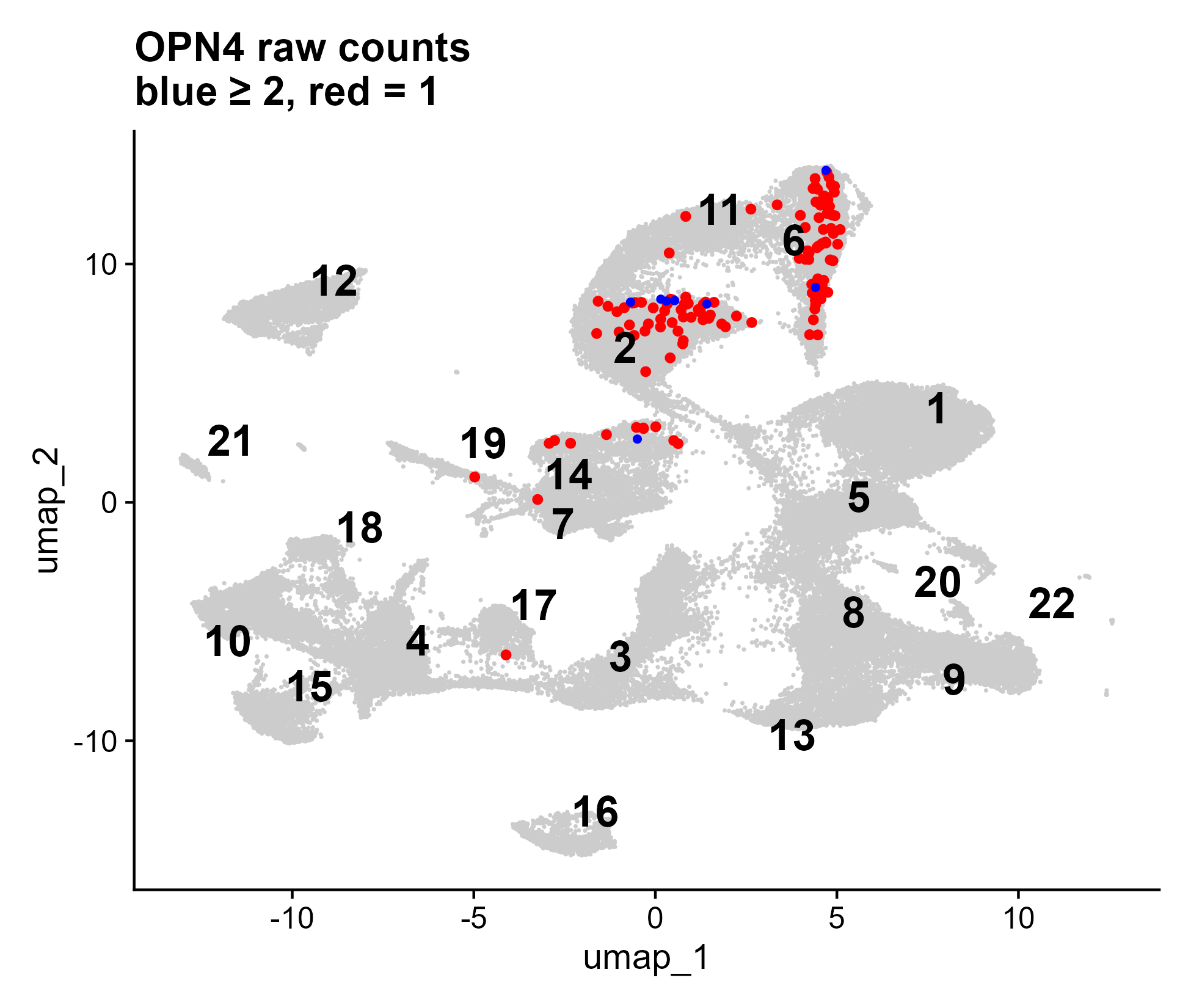


**Figure S12. UMAP visualisation of OPN4-expressing cells in the full integrated RGC-enriched retinal organoid dataset.** Each point represents a single cell; all cells are shown in grey, while OPN4-expressing cells are highlighted. Cells with low OPN4 abundance (SoupX-adjusted RNA count > 0 and < 2) are shown in red, and cells with higher abundance (SoupX-adjusted RNA count ≥ 2) are shown in blue, reflecting relative transcript abundance prior to normalisation.


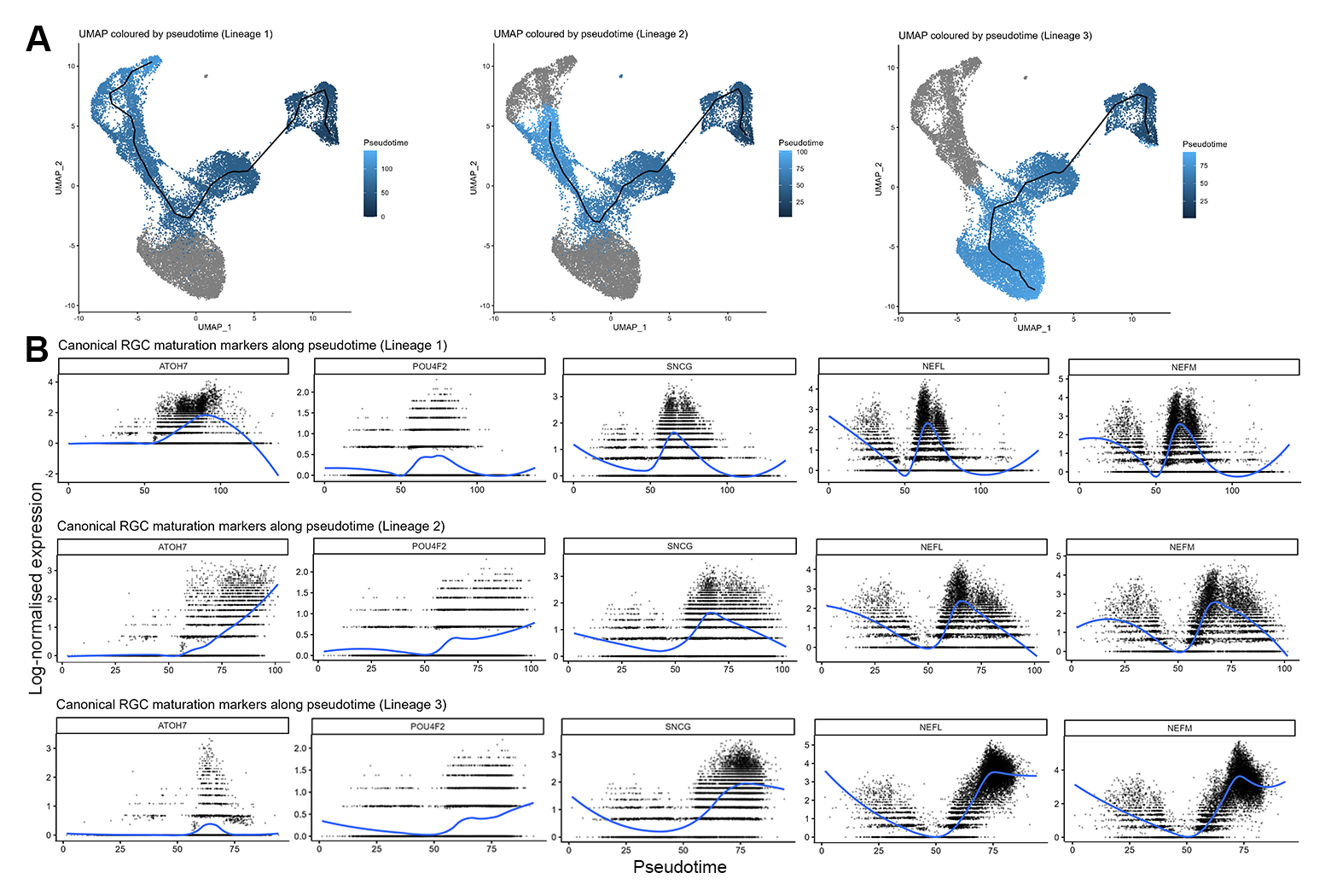


**Figure S13. Pseudotime dynamics of RGC maturation markers.** Slingshot pseudotime trajectories were inferred for the RGC population. (**A**) UMAP visualisation of the RGCs with the three inferred lineages overlaid. Colour represents pseudotime progression, with lighter blue indicating earlier developmental states and darker blue indicating more mature transcriptional states. (**B**) Expression dynamics of canonical RGC maturation markers (*ATOH7*, *POU4F2*, *SNCG*, *NEFL* and *NEFM*) plotted along pseudotime for each lineage. Lineage 1 shows an early RGC differentiation trajectory characterised by transient ATOH7 expression followed by induction of POU4F2 and neuronal markers, consistent with transition from neurogenic progenitors to differentiating RGCs. Lineage 2 represents an intermediate maturation trajectory with progressive activation of RGC markers, whereas Lineage 3 shows minimal ATOH7 and strong neuronal marker expression, consistent with more mature RGC states.


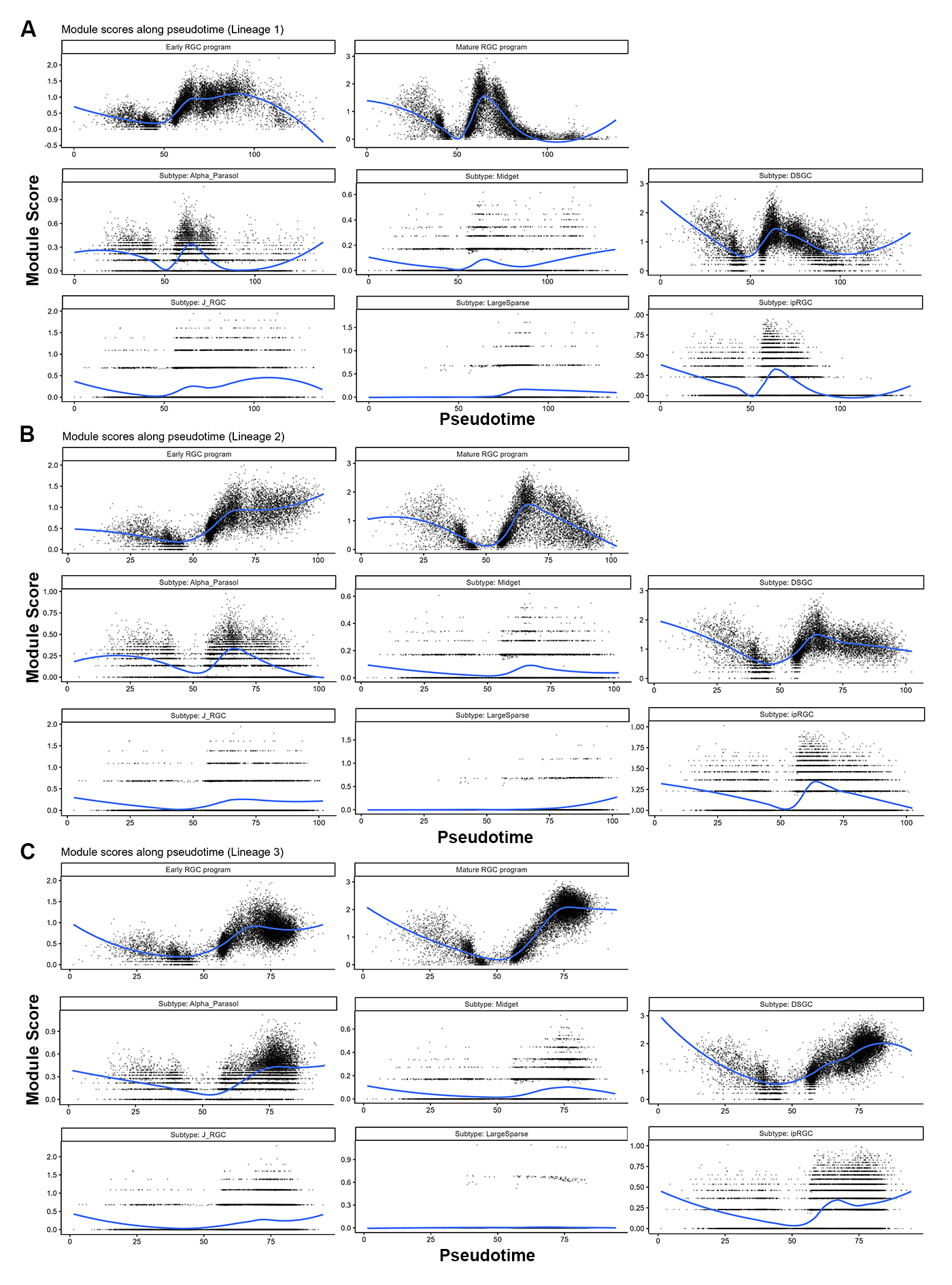


**Figure S14. Pseudotime dynamics of RGC transcriptional programs.** Slingshot pseudotime trajectories showing the module score for early RGC programs, mature RGC programs (Table S6), and subtype-associated transcriptional signatures (Table 2) plotted along pseudotime for lineage 1 (**A**), lineage 2 (**B**), and lineage 3 (**C**). (**A**) Lineage 1 represents an early RGC differentiation trajectory, with transient early neurogenic activity followed by induction of RGC specification and neuronal maturation programs. Subtype-associated programs show weak or transient enrichment, suggesting that subtype specification has not yet been established. (**B**) Lineage 2 reflects an intermediate RGC maturation trajectory, characterised by progressive activation of early RGC and neuronal programs along pseudotime. Transient enrichment of subtype-associated transcriptional signatures suggests the emergence of multiple RGC subtype states during differentiation. (**C**) Lineage 3 represents a late RGC maturation trajectory characterised by strong activation of mature RGC transcriptional programs at higher pseudotime values. Subtype-associated programs, particularly DSGC and Alpha/Parasol signatures, become progressively enriched, consistent with advanced RGC maturation.

**Table S1: Markers used to define early RGC and mature RGC programs derived from human datasets for module score calculation.**

| **Biological stage** | **Genes included** | **References** |
| --- | --- | --- |
| Early developing RGCs | *ATOH7, HES6, DLL3, SOX11, SOX4, NEUROD1, NHLH2, EBF1, RXRG, ELAVL3* | [[1,2]](https://paperpile.com/c/RXybE2/hvAE+stSW) |
| Differentiated RGCs | *RBPMS, POU4F1, POU4F2, ISL1, SNCG, SLC17A6, NEFL, NEFM, GAP43, POU6F2* | [[2–5]](https://paperpile.com/c/RXybE2/snNv+jMl1+CjTE+stSW) |

**Table S2. Summary of cell retention across quality control steps*.*** Number of cells retained after EmptyDrops, SoupX + QC filtering, and doublet removal for each sample, along with the proportion of cells removed at each step.

| **Sample** | **Cells after EmptyDrops** | **Cells After SoupX+QC** | **Cells After Doublet** | **Integrated cells** |
| --- | --- | --- | --- | --- |
| **H9_RGC1** | **19,334** | **18,847** | **16,638** | **16,638** |
| **H9_RGC2** | **23,881** | **23,067** | **19,861** | **19,861** |
| **WAB_RGC2** | **21,678** | **21,140** | **18,926** | **18,926** |
| **WAB_RGC1** | **21,452** | **19,952** | **18,217** | **18,217** |

**Table S3. Distribution of identified cell types across individual datasets and clusters.** Per-cluster cell identities and distribution of cells across H9_RGC1, H9_RGC2, WAB_RGC2, and WAB_RGC1, with number and percentage of cells per dataset. Cluster annotations were assigned based on transcriptional profiles, including canonical markers.

|  |  | H9_RGC1 | | H9_RGC2 | | WAB_RGC2 | | WAB_RGC1 | |
| --- | --- | --- | --- | --- | --- | --- | --- | --- | --- |
| Cluster | Cell identity | # of cells | % of total | # of cells | % of total | # of cells | % of total | # of cells | % of total |
| 1 | Other (HOX-enriched) | 2,127 | 12.79 | 5,947 | 29.96 | 217 | 1.15 | 991 | 5.44 |
| 2 | RGC | 3,560 | 21.4 | 2,047 | 10.31 | 834 | 4.42 | 432 | 2.37 |
| 3 | RGC | 1,086 | 6.53 | 672 | 3.39 | 2,762 | 14.64 | 940 | 5.16 |
| 4 | Retinal progenitor cell | 1,278 | 7.68 | 920 | 4.63 | 487 | 2.58 | 2,247 | 12.35 |
| 5 | Amacrine cell | 1,036 | 6.23 | 1,797 | 9.05 | 682 | 3.61 | 1,339 | 7.36 |
| 6 | Horizontal cell | 1,160 | 6.97 | 688 | 3.47 | 2,271 | 12.04 | 506 | 2.78 |
| 7 | Amacrine cell | 1,495 | 8.99 | 1,886 | 9.5 | 364 | 1.93 | 539 | 2.96 |
| 8 | Photoreceptor- committed cell | 388 | 2.33 | 687 | 3.46 | 2,552 | 13.53 | 531 | 2.92 |
| 9 | Photoreceptor- committed cell | 168 | 1.01 | 1,133 | 5.71 | 2,146 | 11.37 | 661 | 3.63 |
| 10 | Retinal progenitor cell | 462 | 2.78 | 651 | 3.28 | 111 | 0.59 | 2,798 | 15.37 |
| 11 | RGC | 1,346 | 8.09 | 787 | 3.96 | 1,245 | 6.6 | 303 | 1.66 |
| 12 | Other | 21 | 0.13 | 818 | 4.12 | 1,447 | 7.67 | 948 | 5.21 |
| 13 | Photoreceptor- committed cell | 84 | 0.51 | 116 | 0.58 | 2,101 | 11.14 | 94 | 0.52 |
| 14 | RGC | 1,576 | 9.48 | 289 | 1.46 | 345 | 1.83 | 153 | 0.84 |
| 15 | Retinal progenitor cell | 201 | 1.21 | 145 | 0.73 | 392 | 2.08 | 1,230 | 6.76 |
| 16 | RPE | 2 | 0.01 | 81 | 0.41 | 30 | 0.16 | 1,678 | 9.22 |
| 17 | Retinal progenitor cell | 117 | 0.7 | 118 | 0.59 | 263 | 1.39 | 1,104 | 6.07 |
| 18 | Retinal progenitor cell | 184 | 1.11 | 419 | 2.11 | 82 | 0.43 | 698 | 3.83 |
| 19 | Multilineage (stressed) | 276 | 1.66 | 567 | 2.86 | 55 | 0.29 | 472 | 2.59 |
| 20 | Photoreceptor-committed cell | 35 | 0.21 | 72 | 0.36 | 464 | 2.46 | 22 | 0.12 |
| 21 | Retinal progenitor cell | 31 | 0.19 | 9 | 0.05 | 18 | 0.1 | 515 | 2.83 |

**Table S4. Differential abundance of major cell populations.** Differential abundance analysis was performed using binomial generalised linear models (GLM) in R, modelling cell-type counts relative to the total number of cells per sample. The model included cell line (WAB vs H9) and differentiation quality (LessOptimal vs Optimal), defined based on organoid morphology during differentiation. Odds ratios (OR) indicate enrichment (OR > 1) or depletion (OR < 1); values in parentheses represent 95% confidence intervals (CI). False discovery rate (FDR)-adjusted p-values are reported.

| Cell identity | WAB vs H9 OR (95% CI) | FDR | LessOptimal vs Optimal OR (95% CI) | FDR |
| --- | --- | --- | --- | --- |
| Retinal progenitor cell | 0.54 (0.51-0.58) | 1.2E-78 | 11.6 (10.9-12.3) | <1E-300 |
| RGC | 0.84 (0.81-0.87) | 5.6E-19 | 0.30 (0.28-0.31) | <1E-300 |
| Amacrine cell | 0.29 (0.27-0.31) | 3.4E-284 | 1.96 (1.81-2.12) | 3.3E-63 |
| Horizontal cell | 2.56 (2.41-2.73) | 8.2E-182 | 0.21 (0.19-0.23) | 4.9E-212 |
| Photoreceptor- committed cell | 7.88 (7.51-8.28) | <1E-300 | 0.12 (0.12-0.13) | <1E-300 |
| RPE | 0.70 (0.46-1.06) | 0.092 | 63.8 (44.4-91.6) | 4.7E-112 |
| Multilineage (stressed) | 0.12 (0.09-0.16) | 1.1E-50 | 9.11 (6.88-12.0) | 7.0E-54 |
| Other (HOX-enriched) | 0.041 (0.036-0.047) | <1E-300 | 4.95 (4.27-5.74) | 6.5E-99 |
| Other | 3.53 (3.23-3.85) | 2.1E-177 | 0.66 (0.61-0.72) | 9.9E-22 |

**Table S5. Percentage correspondence between global clusters and RGC subclusters.** Row-wise percentages showing the relationship between clusters identified in the global organoid dataset (resolution 0.5; Fig. 4A) and subclusters obtained after RGC-specific reclustering using Harmony-corrected embeddings (resolution 0.3; Fig. 4B). Rows represent the original cluster identities from the full dataset, while columns represent the RGC subclusters identified after subsetting and reclustering the RGC population. Values indicate the percentage of cells from each global cluster that are assigned to each RGC subcluster. Percentages are calculated within each row, so values across each row sum to approximately 100%.

| Global cluster (Fig4A) | Sub1 | Sub2 | Sub3 | Sub4 | Sub5 | Sub6 | Sub7 |
| --- | --- | --- | --- | --- | --- | --- | --- |
| 2 | 93.7 | 0.1 | 0.2 | 0.1 | 0.0 | 5.9 | 0.0 |
| 3 | 1.2 | 2.4 | 49.2 | 0.0 | 39.2 | 7.0 | 1.0 |
| 11 | 0.5 | 82.1 | 0.1 | 0.1 | 0.1 | 17.2 | 0.0 |
| 14 | 0.3 | 1.0 | 0.0 | 98.6 | 0.0 | 0.0 | 0.1 |

**Table S6. Distribution of *OPN4*-expressing cells across clusters in the full integrated RGC-enriched retinal organoid dataset.** Distribution of cells with detectable *OPN4* expression (raw count > 0) across clusters in the unnormalised, SoupX-adjusted RNA count matrix (n = 73,642). The analysis was conducted prior to RGC-specific subclustering and includes all major retinal and off-target cell populations.

| Cluster ID | Total OPN4+ cells | Count = 1 | Count ≥ 2 |
| --- | --- | --- | --- |
| 2 | 51 | 46 | 5 |
| 6 | 60 | 58 | 2 |
| 7 | 1 | 1 | 0 |
| 11 | 3 | 3 | 0 |
| 14 | 11 | 10 | 1 |
| 17 | 1 | 1 | 0 |
| 19 | 1 | 1 | 0 |
| Total | 128 | 120 | 8 |

**References:**

[1. Sridhar A, Hoshino A, Finkbeiner CR, Chitsazan A, Dai L, Haugan AK, et al. Single-Cell Transcriptomic Comparison of Human Fetal Retina, hPSC-Derived Retinal Organoids, and Long-Term Retinal Cultures. Cell Rep. 2020;30:1644–59.e4.](http://paperpile.com/b/RXybE2/hvAE)

[2. Lu Y, Shiau F, Yi W, Lu S, Wu Q, Pearson JD, et al. Single-Cell Analysis of Human Retina Identifies Evolutionarily Conserved and Species-Specific Mechanisms Controlling Development. Dev Cell. 2020;53:473–91.e9.](http://paperpile.com/b/RXybE2/stSW)

[3. Menon M, Mohammadi S, Davila-Velderrain J, Goods BA, Cadwell TD, Xing Y, et al. Single-cell transcriptomic atlas of the human retina identifies cell types associated with age-related macular degeneration. Nat Commun. 2019;10:4902.](http://paperpile.com/b/RXybE2/snNv)

[4. Lukowski SW, Lo CY, Sharov AA, Nguyen Q, Fang L, Hung SS, et al. A single-cell transcriptome atlas of the adult human retina. EMBO J. 2019;38:e100811.](http://paperpile.com/b/RXybE2/jMl1)

[5. Zuo Z, Cheng X, Ferdous S, Shao J, Li J, Bao Y, et al. Single cell dual-omic atlas of the human developing retina. Nature Communications. 2024;15:6792.](http://paperpile.com/b/RXybE2/CjTE)
